# Supplementary material for: Global patterns of tree density are contingent upon local determinants in the world’s natural forests
Source: Commun Biol. 2023 Jan 13;6:47. doi: 10.1038/s42003-023-04419-8 (PMC9839683; doi:10.1038/s42003-023-04419-8)
Supplement: Supplementary file 2 — Description of Additional Supplementary Files [file 42003_2023_4419_MOESM2_ESM.pdf]

Description of Additional Supplementary Files

File name: Supplementary Data 1
Description: Source data for figure 2.

File name: Supplementary Data 2
Description: Source data for figure 4.
